# Is leptomeningeal dissemination in oligodendroglioma predictable? Evidence from a scoping review

**DOI:** 10.3389/fonc.2026.1757720

**Published:** 2026-06-15

**Authors:** Hanna Veronika Salvotti, Francesco Brigo, Paolo Cipriano Cecchi, Davide Costazza, Vania Pirillo, Luca Zavatto, Francesco Erdini, Andreas Schwarz, Pier Paolo Berti

**Affiliations:** 1 Department of Neurosurgery, Hospital of Bolzano (SABES-ASDAA), Teaching Hospital of Paracelsus Medical University (PMU), Bolzano-Bozen, Italy; 2 Research and Teaching Service (IRTS), Hospital of Bolzano (SABES-ASDAA), Teaching Hospital of Paracelsus Medical University (PMU), Bolzano-Bozen, Italy; 3 Department of Pathology, Hospital of Bolzano (SABES-ASDAA), Teaching Hospital of Paracelsus Medical University (PMU), Bolzano-Bozen, Italy

**Keywords:** leptomeningeal metastasis, neuroaxis MRI, oligodendroglioma, risk features, scoping review

## Abstract

**Introduction:**

Oligodendrogliomas (ODGs) are IDH-mutated gliomas with 1p/19q co-deletion, typically associated with better outcomes than astrocytomas. Symptomatic leptomeningeal dissemination (LMD) is exceptionally rare, and predictors of its occurrence remain poorly defined. This scoping review aimed to synthesize the available literature on LMD in ODG and identify potential clinical, radiological, surgical, and histo-molecular risk markers.

**Methods:**

PubMed/MEDLINE and Embase were systematically searched for studies reporting cases of ODG with LMD. Extracted data included demographics, tumor features, imaging, treatment, molecular markers, and outcomes. Findings were descriptively summarized. An illustrative institutional case was also reviewed.

**Results:**

Of 1719 records screened, 15 studies met inclusion, comprising 48 patients. The mean age at LMD diagnosis was 43.1 years, with male predominance (M: F = 2.4:1). Most patients (39 of 41 patients [85.7%]) had WHO grade 3 ODG at LMD diagnosis. When explicitly stated, LMD was associated with surgical entry into the ventricles (2 of 4 patients [50%]), a Ki-67 index of ≥15% (6 of 7 patients [85.7%]), and contrast enhancement on MRI (4 of 8 patients [75%]). The median time from ODG diagnosis to LMD was 35 months (range 0–274 months). Median survival after LMD was 9.9 months (range 1.8-67.7). Our case reflected these patterns, with 14 years of stable disease followed by rapid decline after LMD onset. Treatments included chemotherapy (23 of 39 patients [58.9%]), radiotherapy (14 of 39 patients [35.9%]), and surgery (12 of 40 patients [30%]), with uncertain benefit.

**Discussion:**

LMD in ODG is rare but associated with poor prognosis. Features such as grade 3 histology, ventricular involvement, surgical entry into ventricles, contrast enhancement, and high Ki-67 may indicate potential risk markers. These findings support long-term whole-neuroaxis MRI surveillance in selected patients.

## 1 Introduction

Oligodendrogliomas (ODGs) are a distinct and relatively uncommon subtype of gliomas, accounting for approximately 2–5% of all primary central nervous system (CNS) tumors (1, 2). They primarily affect adults, with peak incidence between 40 and 60 years of age. According to the 5th edition of the World Health Organization (WHO) Classification of CNS Tumors (2021), ODGs are molecularly defined by the presence of a canonical IDH1 (R132) or IDH2 (R172) mutation together with co-deletion of chromosome arms 1p and 19q (3). Additional molecular features, including TERT promoter mutations and retained ATRX expression, further support the diagnosis and assist in therapeutic decision-making (4).

Leptomeningeal dissemination (LMD) describes the spread of tumor cells along the leptomeninges and within the cerebrospinal fluid (CSF) (5). It represents one of the most devastating complications of CNS tumors, often associated with rapid neurological decline and limited treatment options. With improvements in survival among patients with malignant gliomas, LMD is being reported with increasing frequency, occurring in approximately 4.7% of patients during life and up to 25% at autopsy (6, 7). Within the spectrum of gliomas, the highest incidence is seen in IDH-wildtype glioblastoma (GBM) (8, 9). In contrast, symptomatic LMD in ODGs remains exceptionally rare, with only sporadic case reports and small series available in the literature. As a result, no consensus guidelines for treatment have been established. Current management is largely palliative and focuses on symptom control, typically employing radiotherapy and systemic or intrathecal chemotherapy, which may prolong survival to a median of only 3–6 months (7, 10).

The predisposing potential risk features for LMD in ODG are poorly understood. Tumor location, larger size, and proximity to the ventricular system have been hypothesized to facilitate dissemination (11, 12). For example, in IDH-wildtype GBM, shorter distance from the tumor margin to the ventricles has been associated with earlier onset of LMD (12). However, analogous data for ODG remain extremely limited, precluding a clear understanding of these relationships. At the molecular level, alterations such as CDKN2A/B deletion have been linked to more aggressive behavior in ODG, but their specific association with LMD has not yet been demonstrated (13).

Given the rarity of this complication, the incidence, associated features, and clinical course of LMD in ODG remain largely undefined. To address this gap and prompted by a recent illustrative case from our institution, we conducted a scoping review to synthesize the available evidence. Our objective was to systematically examine clinical, radiological, surgical, and histo-molecular features reported in ODG patients with LMD, with the aim of identifying potential predictive markers that could inform risk stratification, optimize surveillance strategies, and ultimately guide therapeutic decision-making in clinical practice.

## 2 Materials and methods

### 2.1 Review objective and framework

This scoping review aimed to systematically map the available literature on LMD in patients with ODG, focusing on clinical, radiological, surgical, and histo-molecular features potentially associated with this rare phenomenon. A scoping review was selected over a systematic review given the rarity and heterogeneity of reported cases, with the aim of mapping existing evidence and identifying knowledge gaps rather than performing quantitative synthesis.

The review was reported in accordance with the PRISMA-ScR (Preferred Reporting Items for Systematic Reviews and Meta-Analyses extension for Scoping Reviews) guidelines (14).

The research question was structured using the PCC framework recommended by the Joanna Briggs Institute for scoping reviews (15):

- Population (P): Patients of any age with histologically and molecularly confirmed ODG.
- Concept (C): LMD, including clinical presentation, diagnostic strategies, potential associated features (clinical, radiological, surgical, histo-molecular), and treatment.
- Context (C): No geographical or institutional restrictions.

The review protocol was prospectively registered in the Open Science Framework (OSF) (https://doi.org/10.17605/OSF.IO/RFUZK).

### 2.2 Study design and search strategy

A scoping review was performed using a comprehensive search strategy to identify primary studies (case reports, case series, retrospective analyses, clinical studies, reviews, and meta-analyses) reporting on LMD in ODG. Literature research was conducted in PubMed/MEDLINE and Embase between June and August 2025. No restrictions were applied during the initial search phase; however, only studies published from 2016 onward were included to reflect the adoption of the revised WHO CNS tumor classification incorporating molecular criteria (16).

Two reviewers (P.P.B. and H.V.S.) independently screened all records for eligibility in a blinded and parallel fashion. Disagreements were resolved through discussion. Reference lists of included studies were also screened, but no additional eligible publications were identified.

The search strategy combined controlled vocabulary (MeSH terms in PubMed/MEDLINE; Emtree in Embase) with free-text keywords. Boolean operators (AND, OR, NOT) and truncation symbols (asterisks *) were applied to refine the results. The search was intentionally broad and included terms related to oligodendroglioma (e.g., “oligodendroglioma, ” “anaplastic oligodendroglioma, ” “1p/19q codeleted glioma, ” “oligodendroglial tumor”) and leptomeningeal disease (e.g., “leptomeningeal dissemination, ” “cerebrospinal dissemination, ” “drop metastasis, ” “spinal gliomatosis, ” “meningeal carcinomatosis”).

The complete search strategy is provided in the **Supplementary Materials**.

### 2.3 Eligibility criteria

Studies were eligible for inclusion if they met the following criteria (see **Table 1**):

**Table 1:** Inclusion and exclusion criteria.

Domain	Inclusion criteria	Exclusion criteria
Population (P)	• Patients of any age with histolog- ically and molecularly confirmed ODG (WHO grade 2 or 3)	• Non-human studies • Articles in languages other than English • Studies addressing primary lep-tomeningeal ODG (distinct clinical entity)
Concept (C)	• LMD, including: – Clinical presentation – Diagnostic strategies – Potential associated features (clini- cal, radiological, surgical, histo-molecular) – Treatment	• Studies not reporting on LMD in ODG
Context (C)	• Without geographical or institutional restrictions	• None
Study Design	• Original patient data (case re- ports, case series, retrospective analyses, clinical studies, reviews and metanalysis) • Published from 2016 onwards (aligned with WHO CNS classification incorporating molecular criteria)	• Grey literature • Articles published before 2016

LMD, leptomeningeal dissemination; ODG, oligodendroglioma.

- Reported on patients with histologically and molecularly confirmed ODG (WHO grade 2 or 3).
- Documented the occurrence of LMD at any point during the disease course.
- Presented original patient data (case reports, case series, retrospective analyses, clinical studies, reviews, or meta-analyses).

Exclusion criteria were (see **Table 1**):

- Articles published before 2016 (to align with the revised WHO CNS tumor classification incorporating molecular criteria, including 1p/19q co-deletion).
- Non-human studies.
- Articles published in languages other than English.
- Studies addressing *primary leptomeningeal ODG* as a distinct clinical entity.

### 2.4 Study selection process

**Figure 1:**
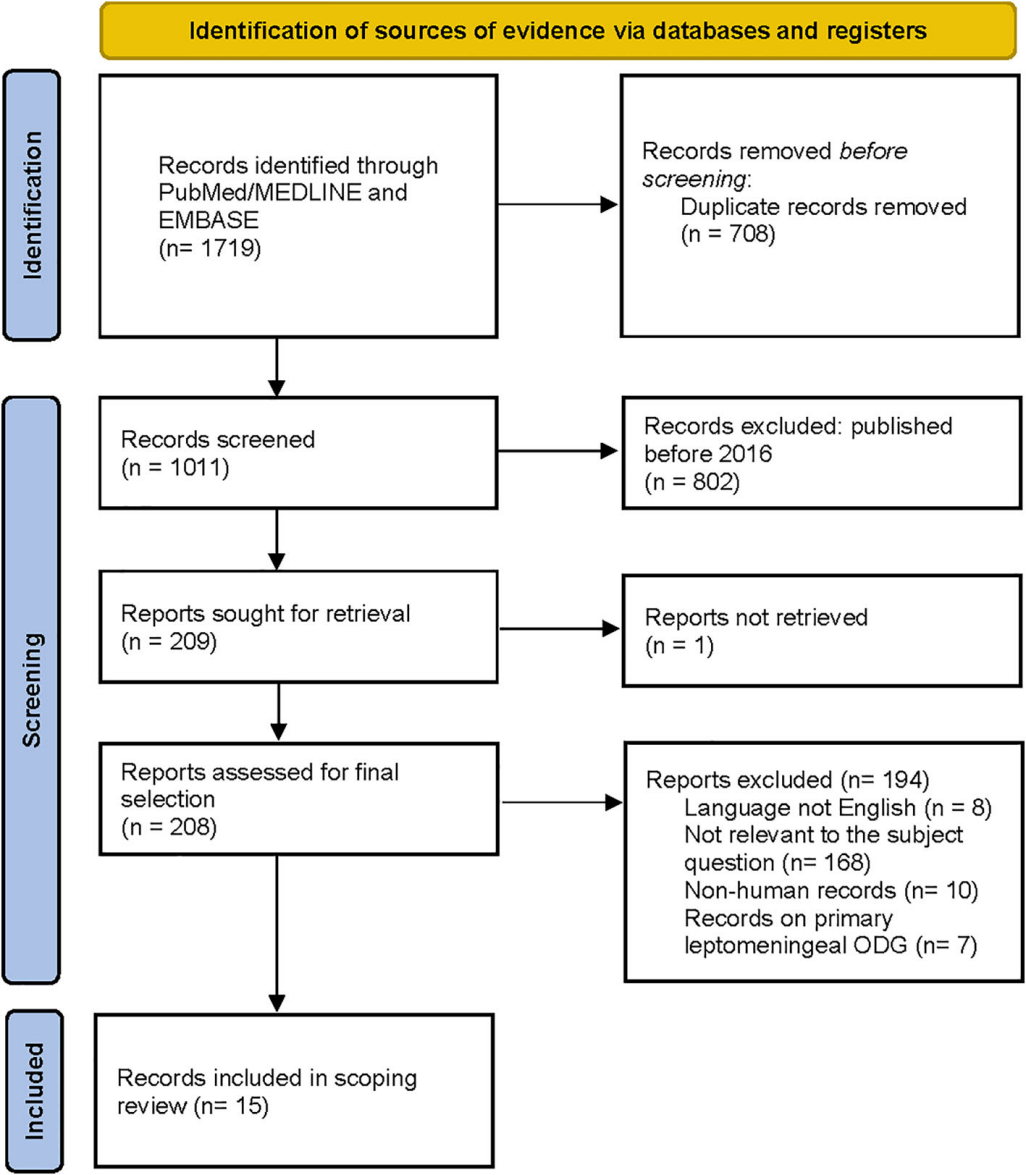
PRISMA-ScR flow diagram showing study selection and exclusion.

All retrieved records were exported to a reference manager, and duplicates were removed. Two reviewers (H.V.S. and P.P.B.) independently screened titles and abstracts. Full-text articles of potentially eligible studies were then assessed against the inclusion and exclusion criteria. Any disagreements were resolved through discussion. The overall selection process is summarized in the PRISMA-ScR flow diagram (**Figure 1**).

### 2.5 Data charting

Data were independently extracted by two reviewers (H.V.S. and P.P.B.) using a pre-specified standardized form. Extracted variables included:

- Study characteristics: Author(s), year, type of publication.
- Patient demographics: Number of patients with ODG grade 2 or 3 at initial diagnosis and at LMD diagnosis; sex distribution; age at LMD diagnosis.
- Clinical characteristics: Karnofsky Performance Status (KPS) at initial diagnosis and at LMD; neurological symptoms.
- Tumor characteristics: Grade (2/3) at initial diagnosis and at LMD; Ki-67 index; additional molecular markers (e.g., CDKN2A/B co-deletion, PDGFR mutation, MYC mutation).
- Radiological characteristics: Tumor side and site; proximity to ventricles (≥1 cm vs <1 cm); surgical cavity communication with ventricles (yes/no); cerebral recurrence before LMD (yes/no); contrast enhancement on MRI (yes/no); radiological pattern (infiltrative/nodular).
- Treatment and outcomes: Use of radiotherapy, chemotherapy, and surgery (reported as percentages).
- Timeline data: Interval from diagnosis to LMD (months); survival after LMD diagnosis (months); overall survival (months).

### 2.6 Data synthesis and reporting

As appropriate for a scoping review, data were synthesized descriptively. Results were summarized in terms of frequencies, ranges, and proportions. Findings were presented in both tabular and narrative formats to highlight recurrent patterns and knowledge gaps. A meta-analysis was not planned, as the available literature on LMD in ODG consists largely of isolated case reports and small case series, lacking the standardized and homogeneous data necessary for quantitative synthesis.

### 2.7 Critical appraisal

As this was a scoping review, no formal risk-of-bias or quality appraisal was performed. Nonetheless, the methodological features and inherent limitations of the included studies -predominantly single-patient case reports and small case series - were acknowledged to contextualize the findings and highlight areas requiring further research.

### 2.8 Case presentation

In addition to the scoping review, we described an illustrative case from our institution. To this end, clinical, radiological, and histo-molecular data were retrieved retrospectively from our institutional patient registry. The patient provided written informed consent for the use of clinical information and imaging for research and publication, in accordance with institutional policies and the principles of the Declaration of Helsinki.

## 3 Results

Two independent reviewers (H.V.S. and P.P.B.) screened all retrieved publications (n = 1719). After removal of duplicates (n = 708), 1011 unique records were available for screening. Of these, 802 were excluded because they were published prior to 2016, i.e., before the adoption of the 4th edition of the WHO CNS tumor classification (16). The remaining 209 records were sought for full-text retrieval.

Following full-text assessment, additional exclusions were applied: articles not published in English (n = 8), studies not directly addressing LMD in ODG (n = 168), non-human research (n = 10), and reports of *primary* leptomeningeal ODG (n = 7). This yielded 15 studies for final inclusion (**Figure 1**, PRISMA-ScR flowchart).

Most included publications were case-based, reflecting the rarity of LMD in ODG. Specifically, the dataset comprised 9 case reports (60%), 1 case series, 1 retrospective review, 1 clinical study, 1 comprehensive analysis, and 2 retrospective cohort studies (**Table 2**). The predominance of single-patient reports highlights the limited availability of systematic data on disease progression in this setting.

**Table 2:** Overview of included studies.

Study ID	Design	N. of included Pt. an sex	Age at LMD	Grading at diagnosis	Grading at LM	Malignant transformation	Ki-67% index	Contrast enhancement in brain MRI	Proximity to ventricle	Surgical opening of ventricle	OS 1st Dx to Death (mo)	OS 1st Dx to LMD (mo)	OS LMD to Death (mo)
Andersen et al. (2019)	Retrospective Review	28 (18M/10F)	44*	G2 (11); G3 (17)	G3 (28)	11	–	–	–	–	132* (8.5–279)	61.7* (3.9–274.8)	10.8* (1.8–67.7)
Andres et al. (2020)	Case Report	1 (M)	47	G3	G3	0	70–80%	1	0	0	10	8	2
Attri et al. (2022)	Case Report	1 (M)	38	G2	G3	1	less than 1%	–	1	1	120^§^ (ATP)	108	12^§^ (ATP)
Burger et al. (2016)	Case Series	1 (M)	33	G3	G3	0	–	–	–	–	–	10	4.6
Carrizosa et al. (2017)	Case Report	1 (M)	46	G2	G3	1	2–3% to 80%	1	1	0	–	24	–
Karaman et al. (2022)	Case Report	1 (F)	55	G3	G3	0	30%	0	1	–	–	36	–
Keskin et al. (2022)	Case Report	1 (M)	52	G2	G2	0	–	1	1	–	51	48	3
Park et al. (2023)†	Comprehensive analysis	3 (–)	–	G2 (1); G3 (2)	–	–	–	–	–	–	–	–	–
Park et al. (2022)†	Retrospective CCS	4 (–)	–	–	–	–	–	–	–	–	–	–	–
Shaghaghian (2023)	Case Report	1 (M)	50	G3	G3	0	–	1	1	–	180^§^	168	12^§^
Sorge et al. (2017)	Case Report	1 (M)	8	G3	G3	0	15%	1	1	1	42^§^	0	42^§^
Terrani et al. (2023)	Case Report	1 (M)	18	G3	G4	1	–	1	1	–	39	34	4
Velz et al. (2019)	Case Report	1 (M)	55	G2	G3	1	–	0	0	–	–	168	–
Im et al. (2020)	Retrospective CCS	1 (F)	47	–	G3	–	–	–	–	–	–	–	–
Kondo et al. (2017)	Clinical Study	2 (M)	43*	–	G3 (2)	–	P1: 75%; P2: 35%	–	–	–	19.2*	9.3*	9.9*
Present Case	Case Report	1 (M)	37	G2	G3	1	5% to 15%	1	1	1	168	167	1

*median; §minimum; LMD, leptomeningeal dissemination; OS, overall survival; Dx, diagnosis; ATP, at time of publication; “–” indicates data not reported or unavailable; † These studies likely include overlapping cohorts.

Notably, two of the included studies originated from the same institution, covered overlapping time periods, and involved largely similar patient cohorts (17, 18). One study reported four ODG patients with LMD (18), while the other described three (17). Given the identical timeframe and authorship, it is highly likely these patients were drawn from the same database. The key distinction is that one study identified a single patient with LMD at the time of ODG diagnosis, whereas the other described two such cases. An overview of the included studies and their key findings is presented in **Table 2**.

Across all publications, a total of 48 patients with ODG and LMD were identified. The mean age at LMD diagnosis was 43.1 years (range: 8–68), with a male predominance (male-to-female ratio: 2.4:1). At initial diagnosis, 33.3% of patients (16/48) had grade 2 ODG and 52.1% (25/48) had grade 3 ODG, while in 7 cases (14.6%) the grade was not reported (18–20). At the time of LMD, 81.2% (39/48) had grade 3 ODG, one patient had grade 2, and in eight cases the grade was unspecified (17, 18). Notably, one controversial case described progression from grade 2 ODG to grade 4 IDH-wildtype GBM, and two cases reported transformation from astrocytoma to ODG (20, 21). In three patients, 1p/19q co-deletion status was not clearly stated (19, 22, 23). Although these works were published after the introduction of 2016 WHO CNS classification, which mandated 1p/19q co-deletion for oligodendroglioma, these studies may reflect transitional adoption of the new criteria and potential misclassification cannot be ruled out.

Where tumor localization was reported (12/48), the most frequent sites were frontal lobe (7 of 12 patients [58.3%]), parietal/parieto-occipital lobe (4 of 12 patients [33.3%]), and temporal/temporo-parietal lobe (3 of 12 patients [25%]). Proximity to the ventricular system was explicitly noted in 7 of 9 cases (77.8%), although no precise distance was provided. Therefore, a tumor was deemed close to the ventricle only when explicitly reported as such by the authors. In 2 of 4 patients, direct communication of the surgical cavity with the ventricles was described (24, 25).

Treatment prior to LMD was reported for 40 patients: 90% (36/40) received adjuvant chemotherapy and 80% (32/40) received radiotherapy. Cerebral recurrence or enlargement of residual ODG before LMD was documented in 40% (4/10) of cases (21, 23, 26, 27).

The interval from initial tumor diagnosis to LMD ranged from 0 months—cases diagnosed with LMD at presentation—to 274.8 months, with a median of 35.0 months. Median survival after LMD diagnosis was 9.9 months (range: 1.8–67.7). Three patients remained alive at last follow-up (12, 42, and 42 months, respectively) (24–26). Overall survival (OS) from initial ODG diagnosis to death was available for 33 patients, with a median OS of 46.5 months (range: 8.5–279).

Clinical presentation of LMD was variable. In the 10 cases where symptoms were detailed, paraparesis or unilateral lower limb paresis were most common (60%), while headache, nausea, or back pain were reported in 30%. The mean Karnofsky Performance Status (KPS) at LMD diagnosis, available for 30 patients, was 81.

MRI was the principal diagnostic tool for LMD. In 20 of 40 cases (50%), spinal MRI was the first investigation prompted by clinical symptoms. Nevertheless, brain MRI was performed in most cases (31/40). Detailed MRI descriptions were available for only eight patients: among these, four demonstrated contrast enhancement at initial diagnosis. In two cases, tumors that were initially non-enhancing recurred with contrast enhancement. No clear differences in growth pattern (infiltrative vs. nodular) were identified.

Where reported (n = 7), a high Ki-67 proliferation index (≥15%) was present in six patients (85.7%) (20, 25, 28–30). One report described a striking increase in Ki-67 from 2–3% at initial diagnosis to 80% at the time of LMD, suggesting markedly increased proliferative activity over the disease course (29).

Treatment strategies varied considerably across cases (**Table 3**). Surgery was performed in 12 of 40 patients (30%), most commonly decompressive laminectomy (n = 7), with three patients also undergoing concurrent leptomeningeal biopsy. Ventriculoperitoneal shunts for obstructive hydrocephalus were placed in four cases, while the surgical procedure was unspecified in one. Adjuvant radiotherapy was administered in 14 of 39 patients (35.8%), and chemotherapy in 23 of 39 (58.9%). Treatment regimen was not specified in nine cases. Among those with documented therapy, four patients were clearly reported to have received combined chemo-radiotherapy, although this number may have been as high as nine when partially documented cases were considered. Six patients (16.2%) received neither chemotherapy nor radiotherapy and were managed with best supportive care.

**Table 3:** Therapeutic approaches.

Study	Surgery	Adjuvant RT	Adjuvant ChT	RT + ChT	Neither RT nor ChT
Andersen et al., 2019	2	5	17	–	5
Andres et al., 2020	1	0	0	0	1
Attri et al., 2022	1	1	1	1	0
Burger et al., 2016	–	1	1	1	0
Carrizosa et al.2017	1	1	1	1	0
Karaman et al., 2022	1	1	0	0	0
Keskin et al., 2022	1	1	0	0	0
Shaghaghian 2023	1	1	1	1	0
Sorge et al., 2017	1	0	1	0	0
Terrani et al., 2023	1	1	0	0	0
Velz et al., 2019	1	–	–	–	–
Kondo et al., 2017	1	2	1	0	0
Total (n=40)	12	14	23	4	6

RT, Radiotherapy; ChT, Chemotherapy; ‘–’ indicates data not reported or unavailable; *only studies where treatment is explicated are reported in this table.

Survival outcomes were inconsistently reported. Where available, the median survival after LMD was 9.9 months for patients treated with radiotherapy and/or chemotherapy. In contrast, the only patient explicitly documented as receiving best supportive care survived two months.

### 3.1 Case presentation

**Figure 2:**
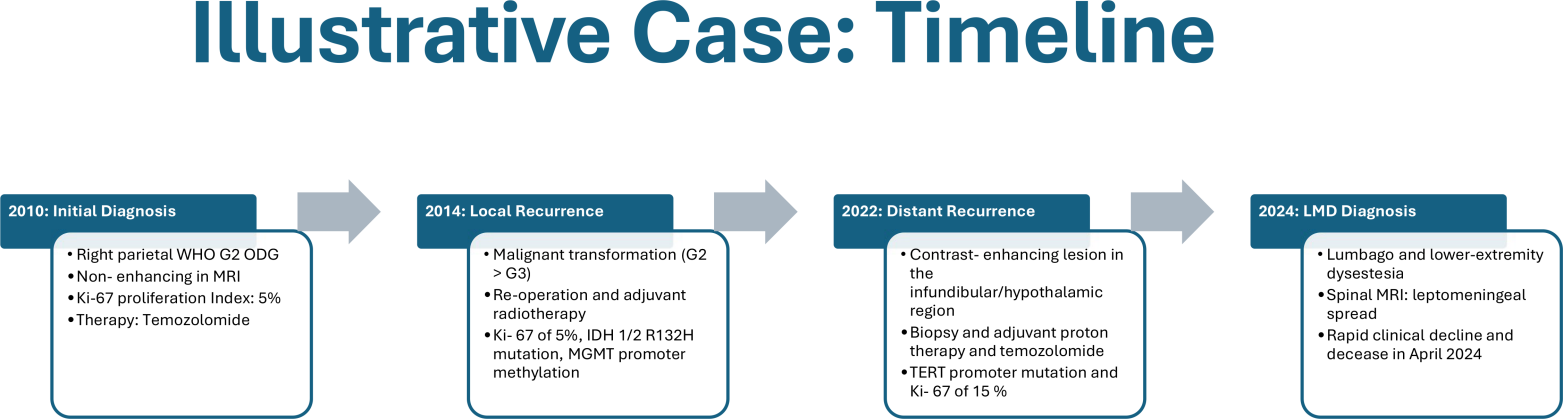
Clinical timeline of the illustrative case including disease progression, interventions and molecular shifts.

To complement the findings of our scoping review, we present the following illustrative case of LMD in ODG, managed at the Neurosurgical Department of our institution (**Table 2**, **Figure 2**).

**Figure 3:**
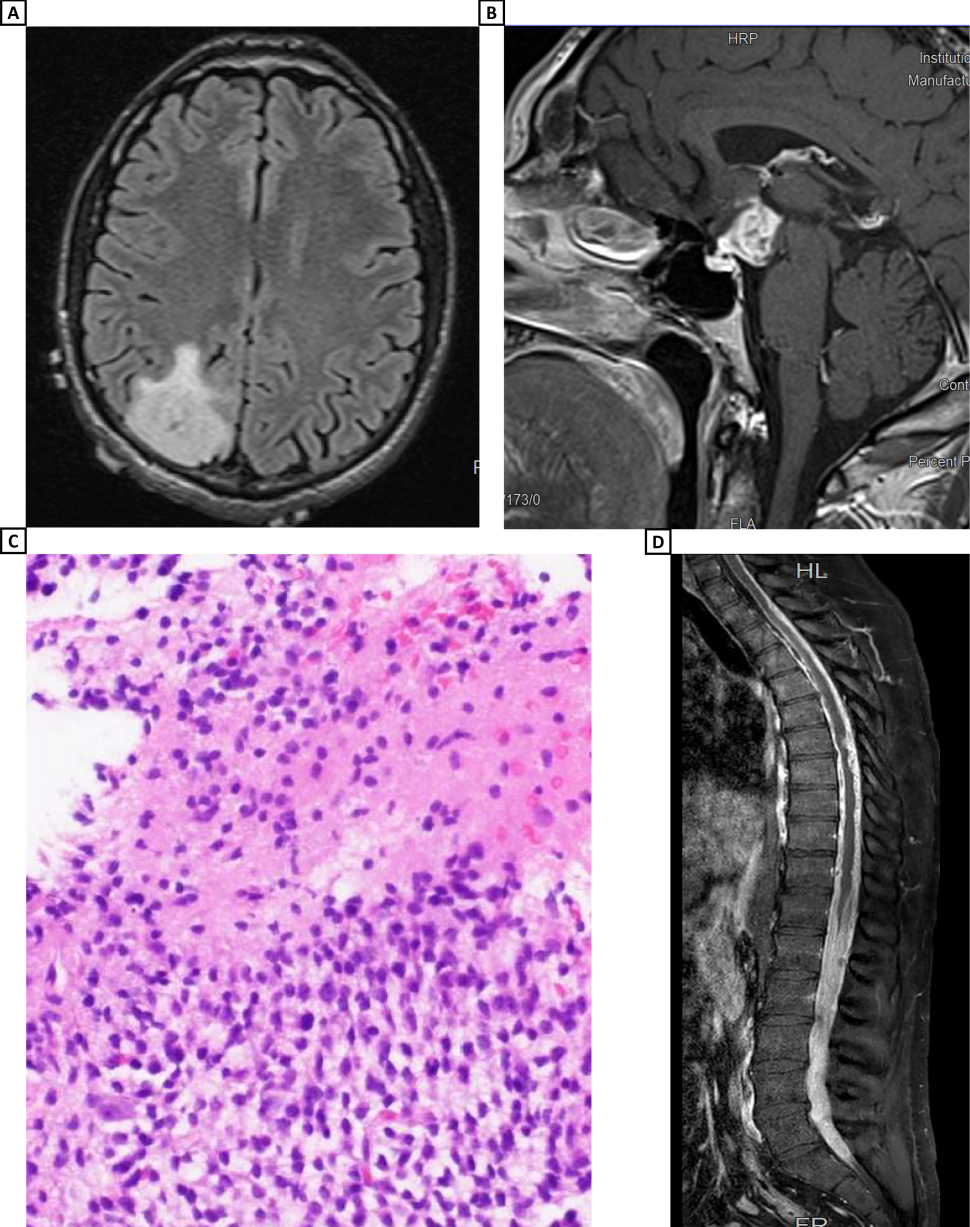
**(A)** Brain MRI at initial diagnosis (2010): FLAIR sequence showing a hyperintense lesion in the right parieto-occipital region. **(B)** Brain MRI at recurrence (2022): T1 post-contrast sequence demonstrating a distal enhancing lesion in the hypothalamic region. **(C)** Histopathology: tumor composed of monomorphic cells with the classic “fried-egg” appearance, arranged in a disorganized pattern within a delicate capillary network. **(D)** Spinal MRI (2024): diffuse leptomeningeal contrast enhancement along the spinal canal, consistent with dissemination.

A 37-year-old male patient was diagnosed in 2010 with a right parietal ODG, WHO grade 2. On MRI, the lesion appeared non-enhancing and homogeneously hyperintense on T2-weighted and FLAIR (Fluid-Attenuated Inversion Recovery) sequences (**Figure 3A**). The Karnofsky Performance Status (KPS) at diagnosis was 100. Histopathological analysis after initial surgical resection confirmed 1p/19q co-deletion and a Ki-67 proliferation index of 5%. Postoperatively, the patient received temozolomide chemotherapy (150–200 mg/m², days 1–5 every 28 days, May 2012–February 2013), which was complicated by poor compliance and reduced bone marrow tolerance.

In 2014, reoperation for local recurrence revealed malignant transformation from grade 2 to grade 3 ODG. Histopathology demonstrated a Ki-67 index of 5%, IDH1/2 R132H mutation, and MGMT promoter methylation. Postoperative radiotherapy was administered.

In 2022, MRI showed a new contrast-enhancing lesion in the infundibular region extending into the right hypothalamic area (**Figure 3B**). Biopsy via right pterional craniotomy confirmed recurrent ODG, with TERT promoter mutation and Ki-67 index of 15% (**Figure 3C**). A third ventriculostomy was performed through the lamina terminalis. No CDKN2A/B co-deletion, PDGFR, or MYC mutations were detected. The patient was treated with proton therapy (total dose 59.4 Gy) and 12 cycles of temozolomide, achieving complete radiological response.

In March 2024, fourteen years after initial diagnosis, the patient presented with lumbago and lower extremity dysesthesia (KPS 80). Brain MRI was unremarkable, but spinal MRI revealed extensive leptomeningeal contrast enhancement along the spinal canal, consistent with dissemination (**Figure 3D**). Rapid neurological decline followed, and despite supportive care, the patient died in April 2024.

## 4 Discussion

In this study, we present an illustrative case of LMD in ODG, together with a scoping review synthesizing clinical, radiological, histopathological, and molecular features of this rare phenomenon. LMD is an exceptionally uncommon complication in gliomas. A large retrospective series from Memorial Sloan Kettering Cancer Center reported an overall incidence of 4.7% in glioma patients (6), while a 2011 population-based study estimated LMD occurrence in ODG at 3.9% (31). However, the latter predates the incorporation of IDH mutation and 1p/19q co-deletion into the WHO diagnostic criteria and therefore included tumors that would not be classified as ODG today. Our review identified only 48 well-documented cases of LMD in ODG. This number likely underestimates the true prevalence, given potential reporting bias, the exclusion of non-indexed or non-English publications, and the likelihood of unpublished cases. Nevertheless, the scarcity of documented reports highlights the rarity of this entity. It is also important to note that our review included only studies published since 2016, reflecting the adoption of molecular classification. Earlier literature largely focused on the correlation between 1p/19q status and LMD, a topic of diminished relevance under the current CNS WHO classification (32).

Although infrequent, the clinical impact of LMD is considerable. It is typically associated with advanced or recurrent disease and portends a poor prognosis (33). Based on our synthesis, median survival following LMD is approximately 10 months. Despite the generally indolent course of ODG, dissemination is followed by rapid neurological deterioration and limited therapeutic options (6, 34). The most frequent presenting symptom was lower extremity paresis, although less specific complaints such as back pain or confusion were also observed (22, 23, 26). This variability underscores the need for standardized diagnostic approaches. Currently, MRI and cerebrospinal fluid cytology remain the principal modalities for diagnosing LMD (35). In our review, MRI was the most frequently employed tool, underlining its critical role in early detection (6, 20–30). These findings suggest that whole-neuroaxis MRI should be strongly considered in ODG patients who develop new neurological symptoms.

The first-line treatment for ODG remains maximal safe surgical resection, typically followed by radiotherapy and chemotherapy—most commonly procarbazine, lomustine, and vincristine (PCV), depending on tumor grade and the presence of high-risk features such as residual disease, advanced age, or midline shift (36, 37). Temozolomide is often used as an alternative owing to its better tolerability and easier administration (38). For non-enhancing grade 2 lesions, anti-IDH therapies have recently emerged as a promising approach (39). A detailed discussion of treatment strategies for primary ODG is beyond the scope of this work; here, we focus specifically on the management of LMD in ODG (40).

Currently, no scientific consensus exists regarding the optimal treatment of leptomeningeal metastases (5). In the studies reviewed, approximately 30% of patients underwent surgery, most often for spinal cord decompression or leptomeningeal biopsy, while ventriculoperitoneal shunts were implanted in four cases. More than one third of patients received radiotherapy, and nearly 60% received chemotherapy. Similar to IDH-wildtype GBM, survival appeared longer in patients treated with radiotherapy and/or chemotherapy compared with those managed with best supportive care. However, the marked clinical and therapeutic heterogeneity, the small number of cases, and incomplete reporting substantially limit the ability to draw robust comparisons or establish definitive survival differences (6, 20–26, 29, 30) Prospective multicenter analyses will be essential to develop evidence-based strategies.

In our illustrative case, overall survival (OS) was 14 years from the time of initial diagnosis, considerably longer than the median OS of 46.5 months observed across the reviewed cohort. The interval from ODG diagnosis to LMD across reviewed studies ranged from 0 to 274.8 months, underscoring the heterogeneity of this patient population. Importantly, these findings highlight that LMD can emerge even after prolonged recurrence-free survival, abruptly worsening the prognosis. This supports the rationale for extended follow-up, including long-term neuroaxis surveillance, particularly in patients with high-risk features.

As emphasized, the primary goal of this review was to explore potential predictive factors for LMD in ODG. While limitations in the current literature posed challenges, we organized our synthesis under four major domains—radiological, surgical, histopathological, and molecular factors (**Table 4**)—to provide a structured discussion of possible risk markers that may guide future surveillance and management strategies.

**Table 4:** Possible associated features of LMD.

Domain	Findings across studies
Tumor Grading	• *De novo* grade 3 tumors were more frequently associated with LMD • Malignant transformation from grade 2 to grade 3 was commonly observed at recurrence, either preceding or coinciding with the onset of LMD
Ki-67 index and other Molecular features	• ODGs typically exhibited a low Ki-67 index (≤ 5%) • Higher Ki-67 values (≥ 15%) were associated with increased tumor aggressiveness • Ki-67 indices ≥ 15% were frequently reported in association with LMD • CDKN2A/B co-deletion, PDGFR alterations, or MYC mutations were inconclusive
Proximity to the Ventricular System	• When explicitly reported, proximity of the primary ODG to the ventricular system was noted in the majority of cases • No exact measurements were provided, only qualitative assessments were reported
Ventricular Opening During Surgery	• When clearly indicated, ventricular opening was reported in approximately 50% of cases • Surgical details were often missing
Contrast Enhancement on MRI	• ODG cases that developed LMD during their natural course frequently exhibited contrast enhancement even at initial diagnosis • Several studies reported initially non-enhancing ODGs developing intense contrast enhancement at recurrence, in association with LMD

LMD, Leptomeningeal dissemination; ODG, Oligodendroglioma.

### 4.1 Radiological factors

Tumor location may play a critical role in the risk of dissemination. A recent study on H3 K27M–mutant gliomas identified proximity to the ventricular system as a significant risk factor for LMD (41). To date, this relationship has not been systematically examined in ODG. In our case, recurrence in the hypothalamic region preceded the diagnosis of LMD. In retrospect, this distal recurrence may have reflected an early tendency toward dissemination through the ventricular system, suggesting a higher risk of subsequent LMD. Similarly, our scoping review identified a high incidence of primary ODGs located near the ventricular system, reinforcing the hypothesis of cerebrospinal fluid–mediated tumor spread (21, 22, 24–26, 29, 30). However, none of the studies specified exact tumor–ventricle distances; a tumor was considered “close” only when explicitly reported by the authors. Dedicated investigations in ODG are warranted to clarify this association, which may hold prognostic and therapeutic significance.

Gliomas are inherently infiltrative, yet distinct radiological patterns have been described—nodular versus infiltrative—based on the tumor’s interface with surrounding brain tissue and the extent of peritumoral edema (42, 43). These imaging characteristics may reflect biological differences. While nodular morphology is occasionally seen in GBM, it is more frequently associated with ODG (43). In our case, the initial grade 2 parietal lesion showed a nodular pattern, whereas the grade 3 hypothalamic recurrence appeared more infiltrative. Unfortunately, none of the included studies addressed radiological growth patterns. Future work should explore whether such imaging phenotypes correlate with tumor aggressiveness or risk of LMD.

Contrast enhancement in ODGs does not always equate to malignancy or progression, but several studies suggest it may signal malignant transformation and highlight biologically aggressive tumor regions (“hot spots”) (44, 45). In our review, 4 of 8 cases demonstrated contrast enhancement at initial diagnosis, and two non-enhancing tumors recurred with enhancement. In our case, the primary parietal lesion was non-enhancing, whereas the hypothalamic recurrence showed marked enhancement. Collectively, these findings support the hypothesis that pronounced contrast enhancement—particularly at recurrence—may be associated with a greater likelihood of LMD development.

### 4.2 Surgical factors

A substantial body of evidence suggests that surgical entry into the ventricular system may facilitate tumor dissemination (24, 25). In our review, when explicitly reported, ventricular entry was associated with LMD in 50% of cases (2/4) (24, 25). Consistent with this, the surgical approach in our case at the time of hypothalamic recurrence - including pterional craniotomy, opening of the skull base cisterns, and third ventriculostomy - may have contributed to subsequent dissemination. Accordingly, whenever technically feasible, careful intraoperative planning to avoid or minimize ventricular entry should be strongly considered.

### 4.3 Histopathological factors

Histological grade appears to play a pivotal role in the development of LMD (46). Although more than 70% of ODGs are initially classified as WHO grade 2, our review found that 52.1% of patients who later developed LMD had grade 3 disease at diagnosis. This suggests that ODGs prone to dissemination may demonstrate aggressive biological behavior from the outset. At the time of LMD diagnosis, however, all but one of the 16 patients initially classified as grade 2 had progressed to grade 3. Thus, even low-grade tumors seem to undergo malignant transformation before dissemination occurs, indicating that grade 2 histology does not preclude LMD (6, 21, 24, 29).

Our illustrative case supports this observation, as malignant progression preceded LMD onset. Taken together, these findings suggest that LMD in ODG arises predominantly in the context of grade 3 disease or transformation to grade 3. Recurrences associated with histological upgrading may therefore warrant heightened vigilance for leptomeningeal spread. Moreover, patients with grade 3 ODG may benefit from more intensive follow-up strategies than those with grade 2 disease, including consideration of routine whole-neuroaxis MRI surveillance.

### 4.4 Molecular factors

Molecular markers may provide valuable insights for risk stratification. In our case, a Ki-67 proliferation index of 15% was observed at recurrence - considerably higher than the average typically reported in ODGs (~5% or lower) (47). A recent multicenter study from the French POLA network identified a prognostic cut-off of 15%, with ODGs exhibiting Ki-67 ≥15% showing significantly shorter overall survival compared to those with lower values (48). Applying this threshold to our review, several reported cases demonstrated elevated Ki-67, supporting its potential role as a biomarker of aggressive disease and risk of LMD (20, 28, 30). Moreover, one study reported a striking increase in Ki-67 from initial diagnosis to the time of LMD, suggesting that rising proliferative activity may herald dissemination (29). Given that ODGs usually display low Ki-67, this marker may represent a cost-effective and reproducible tool for identifying patients at higher risk of LMD. However, only a few studies have explored the relationship between Ki-67 and prognosis in ODG, underscoring the need for further dedicated investigations (49).

In our case, additional molecular alterations—including CDKN2A/B loss, PDGFR, and MYC—were assessed, although none were detected. Notably, none of the studies included in our review evaluated this broader panel simultaneously. Most were published between 2016 and 2021, before the most recent WHO classification of CNS tumors, which emphasizes the importance of such molecular profiling (3). Among these markers, homozygous deletion of CDKN2A/B has emerged as particularly relevant. A recent multicenter cohort of 494 grade 3 ODG patients identified CDKN2A/B loss as an adverse prognostic factor, associated with reduced overall survival (50). To date, however, no study has systematically examined its relationship with LMD in ODG. While CDKN2A/B loss is increasingly recognized as a marker of aggressive disease, its potential role in LMD development remains unexplored and warrants further study.

### 4.5 Limitations of this scoping review

This scoping review has a few limitations. First, the rarity of LMD in ODG restricts the available evidence, which largely consists of case reports and small series, limiting generalizability and precluding quantitative synthesis. This low level of evidence is partly offset by the descriptive aims of our review, consistent with PRISMA-ScR methodology, which does not require formal critical appraisal. Second, although study selection was performed independently by two reviewers with consensus resolution, some degree of subjective bias cannot be excluded, particularly when eligibility depended on poorly defined or inconsistently reported data. Third, despite a broad search strategy across multiple databases, manual reference screening was limited and yielded no additional studies. Relevant reports in grey literature, non-indexed journals, or non-English languages may therefore have been missed. Some definitions used were necessarily arbitrary. For example, “proximity to the ventricular system” was initially defined as within 1 cm, reflecting evidence of tumor infiltration beyond MRI-visible margins (51, 52). However, because none of the included studies provided quantitative distances, this feature was ultimately recorded only when explicitly mentioned by the original authors.

Although we calculated the median and range values for reported survival outcomes, formal survival estimates cannot be reliably derived from this type of data, particularly because some of the studies included in our scoping review reported only aggregated data, precluding accurate case-level median estimation.

Furthermore, the lack of molecular granularity in several included studies needs to be acknowledged. In a case report progression from oligodendroglioma to IDH-mutant astrocytoma was reported (21). This entity likely exhibits distinct molecular behavior compared to classical oligodendroglioma, which may affect the comparability of findings. In addition, in three cases 1p/19q co-deletion was not explicitly reported (19, 22, 23). Despite publication after the 2016 WHO CNS classification, these studies may reflect transitional adoption of mandatory 1p/19q co-deletion criteria and possible misclassification.

Finally, a specific reporting bias should be acknowledged, as the majority of included cases likely represent atypically aggressive presentations of LMD in ODG, potentially further limiting the generalizability of these findings.

## 5 Conclusions

Leptomeningeal dissemination in ODG remains exceptionally rare, with only a limited number of cases reported. The primary aim of this scoping review was to explore potential associated features that could inform surveillance and therapeutic strategies. Our findings suggest that patients with grade 3 ODG at diagnosis or recurrence - especially those with tumors located near the ventricular system, with surgical cavity communication to the ventricles, showing marked contrast enhancement on MRI, and/or exhibiting an elevated Ki-67 proliferation index - may represent a higher-risk subgroup. For these patients, whole-neuroaxis MRI surveillance should be considered during follow-up, even after long recurrence-free intervals.
