# Supplementary material for: Is leptomeningeal dissemination in oligodendroglioma predictable? Evidence from a scoping review
Source: Front Oncol. 2026 Jun 15;16:1757720. doi: 10.3389/fonc.2026.1757720 (PMC13311375; doi:10.3389/fonc.2026.1757720)
Supplement: Supplementary file 1 [file Table1.docx]

**List of Key Words utilized in PUBMED/MEDLINE and EMBASE for Scoping Review Research “Is Leptomeningeal Dissemination in Oligodendroglioma Predictable? Evidence from a Scoping Review“**

1. **PUBMED/MEDLINE RESEARCH (MESH TERMS/FREE TEXT TERMS COMBINATION)**
2. ("Oligodendroglioma"[Mesh] OR oligodendroglioma* OR oligodendroglial tumor*)

AND

("Leptomeningeal metastasis"[Mesh] OR leptomeningeal dissemination OR leptomeningeal spread OR leptomeningeal disease OR cerebrospinal dissemination OR CSF spread)

1. Oligodendroglioma AND leptomeningeal diffusion
2. (oligodendroglioma OR oligodendroglial tumor OR anaplastic oligodendroglioma OR 1p/19q codeleted glioma) AND (leptomeningeal dissemination OR leptomeningeal metastasis OR CSF spread OR leptomeningeal disease)
3. (oligodendroglioma OR anaplastic oligodendroglioma) AND (leptomeningeal spread OR CSF dissemination) AND (MRI OR "spinal MRI" OR "CSF cytology")
4. oligodendroglioma AND spinal metastasis
5. oligodendroglioma AND spine metastasis
6. (oligodendroglioma OR "anaplastic oligodendroglioma" OR "1p/19q codeleted glioma") AND ("spinal metastasis" OR "spinal dissemination" OR "intraspinal metastasis" OR "drop metastasis" OR "leptomeningeal spinal spread" OR "intradural metastasis" OR "spinal cord metastasis" OR "intramedullary metastasis")
7. Oligodendroglioma and spinal dissemination
8. ("oligodendroglioma"[MeSH Terms] OR "oligodendroglioma"[Title/Abstract] OR "anaplastic oligodendroglioma"[Title/Abstract] OR "oligodendroglial tumor"[Title/Abstract]) AND ("leptomeningeal metastasis"[MeSH Terms] OR "leptomeninges dissemination"[Title/Abstract] OR "leptomeningeal spread"[Title/Abstract] OR "meningeal carcinomatosis"[Title/Abstract] OR "CSF dissemination"[Title/Abstract])
9. ("oligodendroglioma"[MeSH Terms] OR "oligodendroglioma"[Title/Abstract] OR "anaplastic oligodendroglioma"[Title/Abstract] OR "oligodendroglial tumor"[Title/Abstract]) AND ("leptomeningeal metastasis"[MeSH Terms] OR "leptomeningeal dissemination"[Title/Abstract] OR "leptomeningeal spread"[Title/Abstract] OR "meningeal carcinomatosis"[Title/Abstract] OR "CSF dissemination"[Title/Abstract])
10. Oligodendrogliomas AND spinal cord metastases
11. Glioma AND Leptomeningeal metastases AND Magnetic resonance imaging AND survival
12. sequential imaging AND spinal gliomatosis AND mri
13. ("Oligodendroglioma/diagnosis"[MeSH] OR "Oligodendroglioma/surgery"[MeSH]) AND ("Spinal Cord Neoplasms/diagnosis"[MeSH] OR "Spinal Cord Neoplasms/surgery"[MeSH])

**2. PUBMED/MEDLINE MESH TERMS RESEARCH**

("Oligodendroglioma"[MeSH Terms] OR oligodendroglioma) AND ("Meningeal Carcinomatosis"[MeSH Terms] OR "leptomeningeal dissemination" OR "leptomeningeal metastasis")

(Oligodendroglioma[MeSH Terms]) AND Meningeal Carcinomatosis[MeSH Terms]

(Oligodendroglioma[MeSH Terms]) AND Leptomeningeal Neoplasms[MeSH Terms]

**3. EMBASE ADVANCED RESEARCH/EMTREE TERMS**

1. (

  'oligodendroglioma'/exp

  OR oligodendroglioma*:ti,ab,kw

  OR 'oligodendroglial tumor*':ti,ab,kw

  OR 'oligodendroglial neoplasm*':ti,ab,kw

  OR 'anaplastic oligodendroglioma*':ti,ab,kw

)

AND

(

  'leptomeningeal metastasis'/exp

  OR 'leptomeningeal carcinomatosis'/exp

  OR 'leptomeningeal dissemination':ti,ab,kw

  OR 'leptomeningeal metastas*':ti,ab,kw

  OR 'leptomeningeal spread':ti,ab,kw

  OR 'CSF dissemination':ti,ab,kw

  OR 'cerebrospinal fluid metastas*':ti,ab,kw

  OR 'neoplastic meningitis':ti,ab,kw

)
